# Development of a multi-task learning framework with gradnorm for precise wound tissue analysis

**DOI:** 10.1371/journal.pone.0340258

**Published:** 2026-02-12

**Authors:** Hyunyoung Kang, Byungho Oh, Solam Lee, YuSung Chu, Jiye Kim, Sejung Yang

**Affiliations:** 1 Department of Medical Informatics and Biostatistics, Yonsei University Wonju College of Medicine, Wonju, Korea; 2 Department of Dermatology, Yonsei University College of Medicine, Seoul, Korea; 3 Department of Dermatology, Yonsei University Wonju College of Medicine, Wonju, Korea; 4 Department of Precision Medicine, Yonsei University Wonju College of Medicine, Wonju, Korea; 5 Department of Plastic and Reconstructive Surgery, Yonsei University Wonju College of Medicine, Wonju, Korea; University of Vigo, SPAIN

## Abstract

Chronic wounds impose a substantial burden on patients and healthcare systems, necessitating accurate qualification of precise wound analysis for effective diagnosis and treatment. Wound size and the proportional composition of internal tissues are critical indicators of healing progression. Traditional segmentation approaches such as Separate Task Learning (STL) suffer from parameter inefficiency, while Multi-Task Learning (MTL), though efficient, often experiences task imbalance that leads to performance degradation in specific tasks. To overcome these challenges, this study proposes WING-MTL (Wound and Wound Tissue Integrated with Gradient Normalization Multi-Task Learning), a novel MTL framework that dynamically balances gradient magnitudes across tasks to enhance accuracy and training stability. Implemented on an Attention-UNet backbone,and incorporates Gradient Normalization to adjust learning gradients in real time, ensuring balanced optimization for wound and wound tissue segmentation tasks. Quantitative evaluations demonstrate that WING-MTL yields statistically significant improvements over STL and outperforms conventional and advanced MTL methods. Analysis of validation loss revealed convergence of both tasks at the same epoch, indicating balanced learning. Furthermore, improved segmentation performance was observed compared to both STL and MTL approaches, particularly for challenging wound tissue types such as slough and epithelium in qualitative analysis. These findings confirm that WING-MTL addresses the task imbalance inherent in MTL frameworks while maintaining parameter efficiency. WING-MTL was evaluated across diverse backbones, including not only UNet-based model but also Resnet and Transformer-based architectures to validate the architectural flexibility of the proposed framework. Consistent performance improvement across these varied architectures in wound tissue segmentation demonstrates the broad applicability. Furthermore, longitudinal analysis of chronic wound patients was conducted to assess the clinical utility of WING-MTL in real-world scenarios. The framework presents a promising and accurate approach for tracking wound healing progression and serves as a potential adjunct for clinical decision-making in chronic wound care.

## Introduction

Chronic wounds afflict a significant portion of the global population, affecting an estimated 1–2% of individuals in developed nations [1]. This places considerable financial strain on the healthcare systems of these nations and adversely affects the quality of life of patients suffering from chronic wounds. In the United States alone, the prevalence of chronic wounds exceeds 6.5 million individuals, with annual costs exceeding $250 billion, of which approximately $25 billion is allocated to wound care and treatment [2]. Despite this substantial burden, providing efficient wound care remains a formidable challenge, even for experienced clinicians [3]. Wound diagnosis traditionally relies on visual inspection by an expert, which is inherently subjective and reliant on the observer’s proficiency [4]. However, the quantification of wound area and tissue composition could enhance wound diagnosis by providing objective metrics. In response to this need, computational advancements have facilitated the development of algorithms for wound and tissue segmentation, offering valuable diagnostic support [5].

The computational aided segmentation in wound field has been studied in two main directions: Wound Segmentation (WS), Wound Tissue Segmentation (WTS). In the field of WS, there are various methods to measure wound area, and the most common method is to measure the width and length of the wound using a ruler [6,7]. However, this approach is challenged by the irregular shapes of wounds, making it difficult to accurately determine the exact area. While early computer-assisted WS studies were proposed for small, limited datasets using image processing techniques [8–11], they have not demonstrated robustness for larger datasets. Recently, advancements in artificial intelligence have enabled the training of deep learning models for the automatic segmentation of chronic wounds using extensive datasets collected across diverse environments. Consequently, this advancement contributes to more robust studies on WS and quantification than were previously possible [12–15].

WTS involves the partitioning of each pixel constituting the wound into skin wound tissue. To achieve accurate diagnosis and evaluation of patients with chronic wounds, it is imperative to concurrently consider the reduction in wound area and alteration in the proportion of wound tissue. This comprehensive approach is pivotal because the severity of the wound is determined not only by its size but also by the composition of various wound tissues within the wound bed. For instance, research has demonstrated a direct correlation between the amount of necrosis, slough, and wound degradation [16]. Additionally, the increase in epithelial tissue and reduction in the size of the epithelial advancing border in the wound may signify the healing process [17]. Therefore, to enhance their effectiveness as diagnostic tools for patients with chronic wounds, artificial intelligence algorithms must possess the capability to simultaneously monitor and quantify changes in both the wound area and the rate of wound tissue change.

WTS, which partitions wound pixels into different tissue types, presents a more complex challenge and has received less research attention compared to wound area segmentation. WTS poses a more formidable challenge compared to wound segmentation and has garnered significantly less attention in research than studies on wound segmentation to date. Notably, previous representative studies on WTS include those conducted by Ramachandram et al. and Chairat et al [18,19]. Ramachandram et al. developed AutoTrace and AutoTissue models utilizing their proprietary Swift Medical wound dataset, comprising 465,187 large-scale image-label pairs, for wound region segmentation and wound tissue segmentation. The model achieved an Intersection over Union (IoU) score of 86.44% for wound segmentation and an average IoU score of 71.92% for segmenting epithelium, granulation, slough, and eschar [18]. And Chairat et al. proposed a U-net (Unet) based model that integrates artificial intelligence algorithms with automatic color and measurement correction calibrated using small dataset with color patches. The algorithm achieved an IoU score of 69.64% for wound segmentation and an average IoU score of 39.76% for segmenting epithelial tissue, granulation tissue, and necrotic tissue [19].

**Fig 1:**
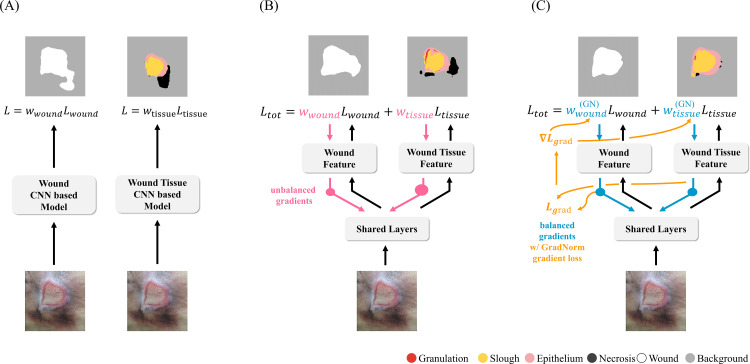
A comparison between deep learning training methods in wound and wound tissue segmentation. (A) Separate-Task Learning (STL), (B) Multi-Task Learning (MTL), and (C) WING-MTL (Wound and Wound Tissue Integrated with Gradient Normalization (GradNorm) Multi-Task Learning). Circle sizes in (B), (C) represent the gradient magnitude of each task. In (B), imbalanced gradient norms across tasks lead to suboptimal training performance within the MTL network. GradNorm, depicted in (C) computes a gradient loss, Lgrad to dynamically adjust loss weights, correcting gradient imbalances and improving MTL process.

In WS and WTS, deep learning approaches such as those illustrated in Fig 1A and 1B [18,19]. The first approach, Separate Task Learning (STL), is a straightforward method but suffers from significant parameter inefficiency, as it requires two separate models for each task. To address the inefficiencies of STL, Multi-Task Learning (MTL) has been proposed as a more efficient alternative [20]. However, MTL approaches share feature representations across tasks to improve parameter efficiency, but this shared learning paradigm can introduce task interference when individual tasks have conflicting optimization objectives or distinct convergence requirements. Previous studies have documented instances where negative transfer between co-learned tasks leads to suboptimal performance for one or more objectives [21]. In our preliminary experiments, MTL exhibited performance degradation in specific wound tissue classes—namely Slough, Epithelium, and Necrosis—when compared to STL as detailed in S1 Table. These observations motivated the development of WING-MTL, which integrates Gradient Normalization (GradNorm) to balance task convergence dynamics and mitigate negative transfer effects. By mitigating task imbalance during knowledge between WS and WTS, WING-MTL retains the parameter efficiency of MTL while delivering consistent performance improvements across STL [22]. Robustness evaluations across various model backbones, including UNet, ResNet, and Transformer architectures confirmed stability of WING-MTL under diverse conditions, with only minor reductions in wound-level performance for certain backbones [23–25]. A comprehensive comparison—including STL, baseline MTL, and advanced MTL methods with WING-MTL —ensures transparent reporting of all methodological insights.

## Materials and methods

### Ethics statement

This study was approved by the Institutional Review Board of Yonsei University Wonju College of Medicine (IRB No. CR324013). All patient data were fully de-identified prior to analysis. Written informed consent was obtained from all participants, and the study was conducted in accordance with the principles of the Declaration of Helsinki.

### Dataset and dataset acquisition

Accurate wound and wound tissue analysis algorithms rely on the precise quantification of both the wound area and tissue composition. Developing an effective deep learning algorithm for wound analysis requires a comprehensive dataset. In this study, wounds were categorized into four distinct tissue types: granulation, slough, epithelium, and necrosis. This section provides a detailed explanation of these wound tissue types and outlines the process for dataset acquisition.

Wound healing is a complex, dynamic process that progresses through four distinct phases: hemostasis, inflammation, proliferation, and remodeling [17]. Each phase plays a critical role in restoring the integrity of wound tissues, and understanding these phases is essential for accurate diagnosis and effective treatment of wounds. The initial hemostasis phase involves vasoconstriction, platelet aggregation, and the formation of a fibrin clot to arrest bleeding. This is followed by fibrinolysis, which dissolves the clot, facilitating the transition to the next phase. During the inflammation phase, immune cells such as neutrophils and macrophages infiltrate the wound site, clearing debris and secreting growth factors essential for wound tissue repair [26,27]. In the proliferation phase, granulation tissue forms, characterized by the deposition of new extracellular matrix (ECM) components by fibroblasts and the migration of keratinocytes to restore the epidermal layer. This phase enhances tissue integrity, marking significant progress in the healing process. Finally, the remodeling phase balances collagen synthesis and degradation, strengthening the newly formed tissue and refining the appearance of the scar to blend with the surrounding skin [28]. Understanding these wound healing stages is crucial because the proportional composition of different wound tissues within the wound bed is a key determinant of the healing stage. For effective diagnosis and treatment, it is essential to quantitatively assess the wound tissue types—such as granulation, slough, epithelial, and necrotic tissues—as they reflect the progression of wound through the healing process.

Granulation tissue is a key indicator of regenerative status, as it supports epithelialization and enables wound coverage. It appears as a rough, moist surface that transitions from pale pink to vibrant red with increasing wound depth, signifying healthy blood supply and progress towards healing [28]. Slough tissue, on the other hand, consists of devitalized material and impedes wound healing. It manifests as yellow or white deposits, often attached to the wound bed, and requires removal to allow the healing process to continue [28,29]. Epithelial tissue emerges during the re-epithelialization phase, marking the wound’s progress towards closure [26]. It forms a protective barrier over the wound, essential for restoring skin integrity [25]. In contrast, necrotic tissue represents dead tissue that hinders healing and necessitates debridement for the restoration of healthy wound tissue [27,30].

Accurately identifying and quantifying wound tissue types through algorithms is crucial in advancing wound healing, as it directly addresses Tissue management and Epithelialization, two key indicators within the TIME (Tissue management, Inflammation and infection control, Moisture balance, and Epithelial edge advancement) framework, which are critical for determining the stage of wound diagnosis [30]. By precisely segmenting and quantifying the proportion of each wound tissue type, the algorithm enables clinicians to monitor wound healing progress more effectively and make informed decisions regarding treatment strategies. The ability to accurately measure these tissue ratios is indispensable for evaluating the efficacy of interventions and ensuring that wounds progress through the necessary stages of healing. The dataset used in this study was compiled from wound images obtained from the Department of Dermatology and Department of Plastic Surgery at Yonsei University Wonju College of Medicine, spanning the period from 2012 to 2020. All wounds were documented using digital cameras, including models from Samsung, Olympus, and Canon (specifically, Canon EOS 700D and Canon EOS 850D). From this extensive dataset, a subset comprising 194 patients diagnosed with chronic wounds such as pressure ulcers and vascular ulcers.

**Fig 2:**
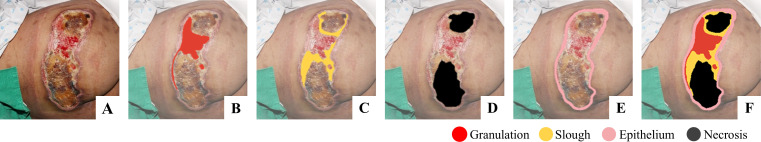
Wound tissue types. **(A)** wound without wound tissue labeling. **(B)** wound with granulation tissue labeling as red. **(C)** wound with slough tissue as yellow. **(D)** wound with necrotic tissue labeling as black. **(E)** wound with epithelium tissue labeling as pink. **(F)** wound with four types of wound tissue labeling.

Physicians manually labeled the dataset into four different types of tissues: granulation tissue, slough tissue, epithelium tissue, and necrotic tissue, defining the wound area as the total area composed of these tissues. The dataset was divided into training, validation, and test sets with 250, 50, and 50 images, respectively, on a patient-to-patient basis. Fig 2 illustrates an example of the dataset and its annotation process. Annotation was facilitated using a self-developed graphical user interface program and Adobe Photoshop. S1 Fig demonstrates an example of labeling performed using the program.

### Configuration

The Convolutional Neural Network (CNN) models were trained with 11GB NVIDIA RTX 2080Ti, using an Adam optimizer with a learning rate of 1 × 10^−4^ and a batch size of 64 for 200 epochs, early stopping with early stopping applied based on validation loss, using a patience of 30 epochs. In addition, the classification CNN model was trained using the sum of cross-entropy loss and Dice loss function, and the regression CNN model was trained using the mean square error loss function. Model development and validation were implemented using the Pytorch framework.

### Architecture of WING-MTL

The primary objective of this study is to improve generalization and segmentation accuracy in multi-task wound analysis by leveraging shared representations between tasks. However, a fundamental challenge in MTL lies in task imbalance, where tasks such as WS and WTS exhibit different convergence behaviors during training. Specifically, WS, a binary classification task distinguishing wounds from the background, converges relatively quickly to optimal parameters. In contrast, WTS, which classifies each pixel into a background or one of four categories, converges more slowly. These discrepancies necessitate a training scheme that dynamically balances learning difficulty between tasks, as empirically observed in the validation loss trajectories presented in discussion section.

To address this, the proposed WING-MTL model incorporates GradNorm, an adaptive gradient normalization algorithm. The GradNorm algorithm adjusts task-specific weights based on gradients from their respective loss functions. Given the varied convergence rates across tasks, the GradNorm algorithm normalizes gradient norms to compare their relative magnitudes, then dynamically adjusts these norms to ensure each task progresses at a comparable rate during training [22]. The hyper parameter α, which influences the strength of normalization effect, was selected to 2.0 empirically shown in S2 Fig.

**Fig 3:**
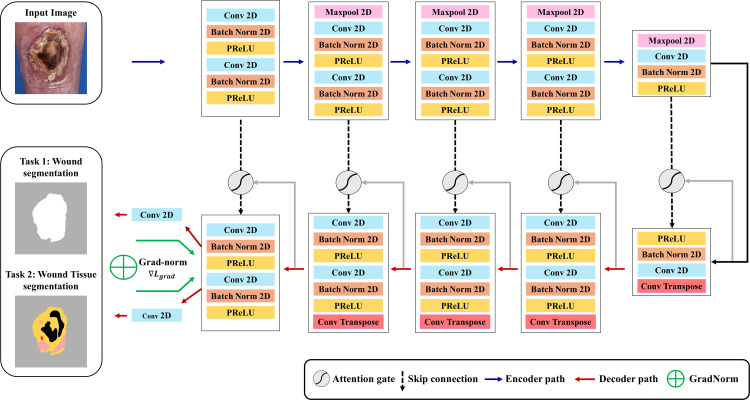
WING-MTL: A Convolutional Neural Network (CNN)-based model enhanced with an attention gate, utilizing MTL with a hard parameter sharing output layer for wound segmentation and wound tissue segmentation. Gradient Normalization (GradNorm) is applied to balance learning difficulty across tasks.

Grounded in the rationale outlined above, the architecture of WING-MTL is systematically described in the following section. The model architecture is constructed utilizing MTL hard parameter sharing, the main approach of MTL, which shares layers among tasks while each task has its own output head, as shown in Fig 1C [20]. To ensure that the observed performance is solely attributable to the structural advantages of MTL and the task balancing mechanism of GradNorm, the model was trained entirely from scratch with randomly initialized parameters. Specifically, no pretrained encoders or weights were utilized, and the model was optimized exclusively on the wound dataset without reliance on any external datasets or transfer learning techniques. The proposed attention-based WING-MTL model, incorporating GradNorm to dynamically balance task-specific gradient magnitudes, is illustrated in Fig 3.

The base model was adapted as a framework emphasizing the Region of Interest (RoI) through the integration of attention blocks into the CNN based model [31]. UNet-based architectures have established themselves as the gold standard for medical image segmentation tasks, demonstrating exceptional effectiveness across diverse biomedical applications. To identify the optimal backbone for WING-MTL, we systematically evaluated three representative UNet variants: the original UNet, EfficientNet UNet, and Attention UNet [31–33]. Following comprehensive evaluation of our wound dataset, Attention UNet demonstrated superior segmentation performance and was consequently selected as the backbone architecture, and the results are summarized in S2 Table. To further assess the robustness and generalizability of WING-MTL, we additionally evaluated its performance with ResNet-based (DeepLabv3) and Transformer-based (SegFormer) backbones. The detailed comparative results are discussed in the results section, Robustness of WING-MTL across backbone models [34,35]. To ensure consistent evaluation across architectures, the GradNorm hyperparameter α was fixed at 2.0 for all WING-MTL implementations, including those using UNet, DeepLabv3, and SegFormer backbones. While task-specific tuning of α may further improve performance, a uniform setting was adopted to isolate the effects of the backbone architecture and maintain methodological consistency.

As part of the evaluation protocol, to evaluate the consistency and output coherence between the predicted WTS and the WS, we calculated the Dice similarity coefficient between the binarized sum of the WTS output and the WS result, as obtained from the WING-MTL model. Specifically, the WTS predictions were aggregated across all tissue classes and binarized to form a unified wound area, which was then compared to the WS output. For the STL setting, in which WS and WTS are modeled independently, an identical procedure was employed: the binarized WTS output was compared against the separately predicted WS output to compute Dice similarity. This analysis quantitatively evaluates the spatial alignment between the two segmentation tasks, presented in result section, Qualitative comparison of segmentation performance between STL and WING-MTL

### Evaluation metrics

A confusion matrix is a critical tool for evaluating the performance of a classification model by illustrating the relationship between predicted and actual classes. It consists of four key elements: True Positives (TP) and True Negatives (TN), which denote correct predictions for the positive and negative classes, respectively, and False Positives (FP) and False Negatives (FN), which represent incorrect predictions, also known as Type I and Type II errors, respectively.

Dice score is a critical metric in medical image segmentation that quantitatively measures the overlap between predicted and ground truth regions. It is particularly effective for datasets with class imbalance, common in medical imaging, and plays a pivotal role in multi-class segmentation tasks by reflecting accuracy across different classes. Consequently, Dice score is employed as the main performance metric in this study, as defined in Equation (1). Sensitivity represents the proportion of true positive pixels correctly identified by the model out of all actual positive pixels. This metric is essential in medical applications where minimizing false negatives is critical for accurate diagnosis and treatment planning. Sensitivity is formally expressed in Equation (2). Precision quantifies the proportion of predicted positive pixels that are truly positive, thereby emphasizing the reduction of false positives. This is vital in medical segmentation to avoid over-diagnosis and unnecessary interventions, enhancing the reliability of model predictions. Precision is detailed in Equation (3).

Each metric is assessed on a per-class basis. For WTS, index ranges from *i = 0* to *i = 4*, representing various wound tissue types: background, granulation, slough, epithelium, necrosis. For wound segmentation, the indices were defined as *i = 0* to *i = 1*, distinguishing between the background and wound.


$$\begin{array}{c} Dice\;score=\frac{\text{2}\times {TP}_{i}}{\text{2}\times {TP}_{i}+{FP}_{i}+{FN}_{i}} \end{array}$$ (1)

$$\begin{array}{c} Precision=\frac{{TP}_{i}}{{TP}_{i}+FP_{i}} \end{array}$$ (2)

$$\begin{array}{c} Sensitivity=\frac{{TP}_{i}}{{TP}_{i}+{FN}_{i}} \end{array}$$ (3)

Confidence intervals were derived based on performance distributions obtained from five independent training repetitions (n = 5). For each class and evaluation metric, the standard deviation across these repeated trials was used to estimate the standard error of the mean, and a two-tailed t-distribution with 4 degrees of freedom (df = n – 1) was applied to compute the corresponding intervals. For statistical analysis, a paired t-test was applied to evaluate differences under the assumption of normal distribution, while the Wilcoxon signed-rank test was used as a non-parametric alternative when normality could not be guaranteed. Statistical significance was defined as p < 0.05, with p < 0.01 considered highly significant [36,37].

## Result

### Quantitative comparison of segmentation performance between STL and WING-MTL

**Fig 4:**
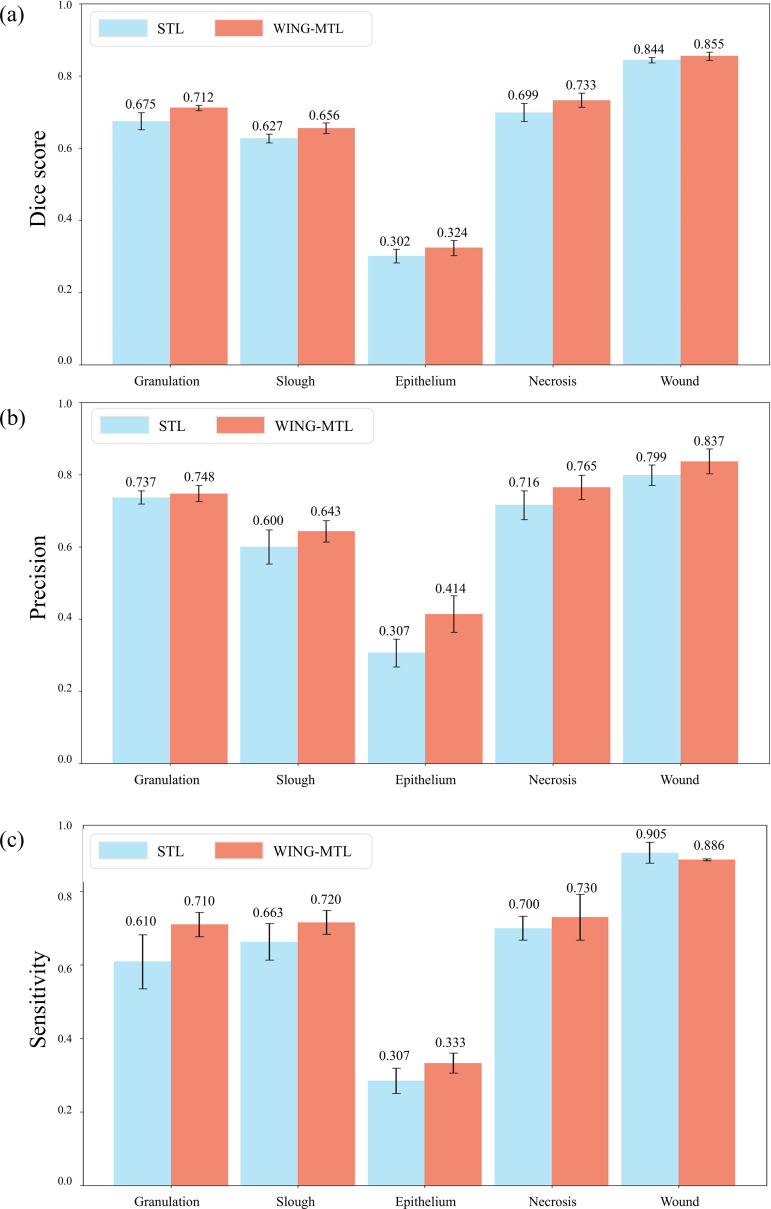
Segmentation performance comparison for wound segmentation and wound tissue segmentation between STL and WING-MTL. (A). Dice scores for STL and WING-MTL models. (B). Precision values for STL and WING-MTL. (C). Sensitivity values for STL and WING-MTL.

The segmentation quantitative performance is examined by comparing WING-MTL and STL. Fig 4 presents the evaluation for WING -MTL over STL across various wound tissue tasks, and wound task in Dice score, precision, and sensitivity values. The comparative analysis of STL, MTL, and WING-MTL methods is presented in S3 Table.

Fig 4 shows the comparative performance of WING-MTL and STL across Dice score, precision, and sensitivity metrics. The results for Dice score in Fig 4A are as follows. The application of WING-MTL to the model yielded the following values: granulation at 0.712 (95% Confidence Interval (CI), 0.706–0.718), slough 0.656 (95% CI, 0.6440.668), epithelium 0.324 (95% CI, 0.307−0.341), necrosis 0.733 (95% CI, 0.7170.749), wound 0.855(95% CI, 0.846–0.865). However, STL shows granulation at 0.675(95% CI, 0.656–0.694), slough at 0.627(95% CI, 0.617–0.637), epithelium at 0.302(95% CI, 0.284–0.317), necrosis at 0.699(95% CI, 0.679–0.719), Wound 0.844(95% CI,0.838–0.850). Although some confidence intervals overlap, statistical analysis confirms that WING-MTL achieves significant improvements over STL across all classes (S3 Table).

The precision results shown in Fig 4B are outlined below. For WING-MTL, granulation yielded 0.748 (95% CI, 0.730–0.766), slough 0.643(95% CI, 0.606–0.680), epithelium 0.414(95% CI, 0.373–0.454), necrosis 0.765(95% CI, 0.738–0.792), wound 0.837(95% CI,0.809–0.865). Conversely, STL shows granulation at 0.737(95% CI, 0.718–0.756), slough at 0.600(95% CI, 0.562–0.638), epithelium 0.307(95% CI, 0.276–0.338), necrosis at 0.699(95% CI, 0.6840.748), wound 0.799(95% CI,0.776–0.822).

The sensitivity result shown in Fig 4C are outlined below. For WING-MTL For WING-MTL, granulation yielded 0.710 (95% CI, 0.684–0.736), slough 0.720 (95% CI, 0.692–0.741), epithelium 0.333 (95% CI, 0.313–0.354), necrosis 0.730 (95% CI, 0.682–0.778), and wound 0.886 (95% CI, 0.884–0.889). Conversely, STL shows granulation at 0.610 (95% CI, 0.553–0.665), slough at 0.663 (95% CI, 0.625–0.701), epithelium at 0.307 (95% CI, 0.259–0.311), necrosis at 0.700 (95% CI, 0.675–0.725), and wound at 0.905 (95% CI, 0.883–0.928). STL yielded higher sensitivity in wound segmentation, which can be attributed to the intrinsic property of sensitivity to reward over-segmentation; a more detailed examination of this phenomenon is presented in discussion section.

### Qualitative comparison of segmentation performance between STL, MTL and WING-MTL

**Fig 5:**
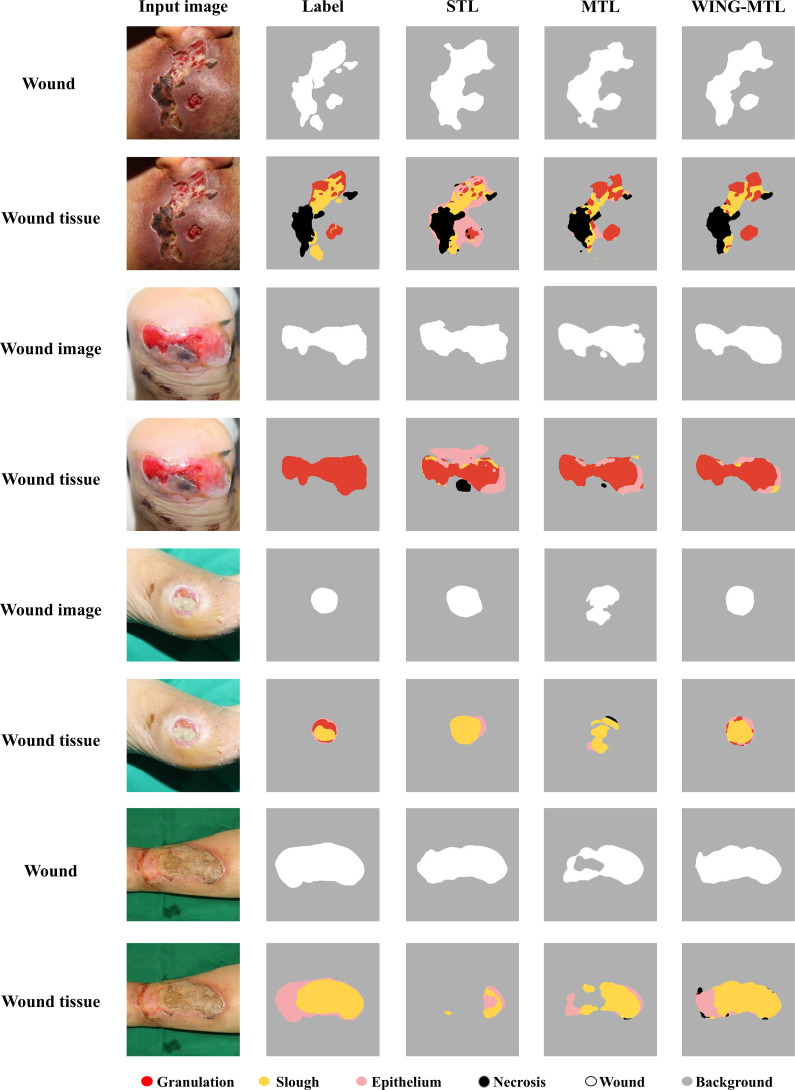
Qualitative comparison between STL, MTL and WING-MTL.

The qualitative results of WS and WTS are presented in Fig 5, with example images of four patients. In each input image, the first row displays the output images for WS task, while the second row presents the output images for WTS task. The first column in each row contains the input image, the second column shows the label, the third column presents the segmentation output for STL, and the fourth column shows the segmentation results from the WING-MTL.

**Fig 6:**
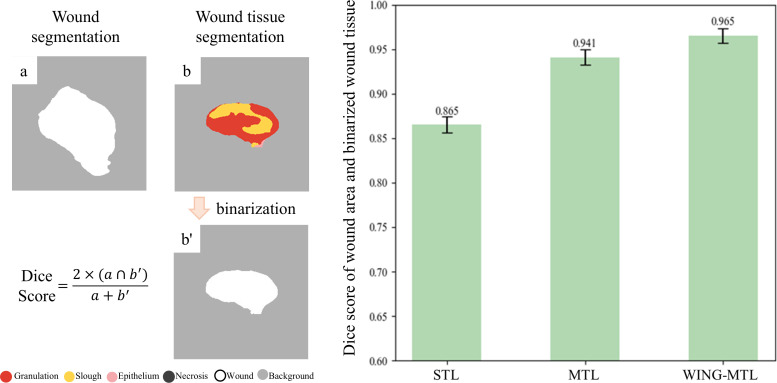
Alignment between the wound area and the binarized aggregated sum of the wound tissue. Dice score was calculated to measure the similarity between the binarized wound tissue segmentation results (b’) and the wound segmentation results (a) for each method. The comparison demonstrates a strong concordance between the wound area and the sum of the wound tissue segmentations in the WING-MTL framework.

The qualitative evaluation revealed that traditional STL methods exhibited discrepancies between the outputs of the WS model and the WTS model. Although the MTL approach showed greater alignment than STL, it still did not achieve perfect consistency—when WS performance suffered, WTS performance was similarly limited. In contrast, the WING-MTL framework demonstrated that the predicted total area of wound tissue closely aligned with the actual wound image, indicating complementary results between wound segmentation and wound tissue segmentation throughout the learning process. To quantitatively assess the consistency between WS and WTS outputs through Dice coefficient between the predicted WS masks and the binarized sum of all WTS classes. As shown in Fig 6, the scores were 0.865 for STL, 0.941 for MTL, and 0.965 for WING-MTL. These results demonstrate that the proposed WING-MTL framework achieves the highest degree of consistency between WS and WTS predictions.

Qualitative analysis was performed to compare the segmentation performance of STL, MTL, and WING-MTL. In the first and second cases, WING-MTL achieved the highest accuracy in WS, whereas STL exhibited over-segmentation of non-wound regions as epithelial tissue in WTS. Although MTL partially mitigated over-segmentation, noise and background classification errors persisted in WTS when WS performance declined.

In the third case, characterized by a high slough ratio but clear boundaries, both STL and WING-MTL accurately segmented the wound and slough tissue. In contrast, MTL produced incomplete WS leading to corresponding WTS performance degradation, indicative of negative transfer between tasks.

The fourth patient case presented segmentation-challenging conditions due to high prevalence of slough and minimal contrast between wound and surrounding skin tones. STL successfully segmented WS but failed to identify slough accurately in WTS, misclassifying or omitting slough regions as background. Joint training with MTL improved WTS performance to some extent yet boundary delineation remained imperfect, leading to misclassification or omission of certain slough areas. In contrast, WING-MTL preserved STL-level accuracy for WS and, by employing GradNorm-based adaptive weighting, delivered precise boundary delineation and markedly improved WTS performance.

These results underscore ability of WING-MTL to maintain optimal inter-task balance through effective knowledge sharing, thereby enabling precise segmentation even in challenging scenarios. Its interactive learning process enhances segmentation granularity, particularly when distinctions between wound and wound tissue are ambiguous, demonstrating robustness that surpasses traditional STL and standard MTL frameworks.

### Quantitative comparison of MTL methods

The effectiveness of the proposed WING-MTL framework was evaluated through comparative analyses against two representative MTL-based methods in Table 1: a conventional MTL, and Nash-MTL, which employs a game-theoretic loss balancing strategy. Nash-MTL formulates multi-task optimization as a multi-agent game, where each task is treated as a player and the optimization seeks a Nash equilibrium among task-specific gradients [38]. Both methods were implemented under identical architectural and training conditions to ensure a fair comparison with WING-MTL. Based on the average Dice scores from five repeated experiments, WING-MTL demonstrated superior performance across all tissue classes compared to both MTL and Nash-MTL. In wound segmentation, however, all three methods achieved comparable Dice scores, showing minimal performance differences. S4 Table provides additional performance metrics including sensitivity and precision. These results confirm that the proposed WING-MTL achieves relatively higher accuracy than the baseline MTL and Nash-MTL methods.

**Table 1:** Quantitative comparison of Dice score among MTL methods.

	Granulation	Slough	Epithelium	Necrosis	Wound
MTL	0.703 (0.684-0.709)	0.620 (0.604-0.656)	0.296 (0.252-0.340)	0.694 (0.673-0.714)	0.851 (0.845-0.857)
Nash-MTL	0.686 (0.674-0.707)	0.620 (0.599-0.629)	0.290 (0.256-0.326)	0.662 (0.646-0.709)	0.853 (0.838-0.872)
WING-MTL	**0.712** (0.706-0.718)	**0.655** (0.644-0.668)	**0.324** (0.307-0.341)	**0.733** (0.717-0.749)	**0.856** (0.846-0.865)

### Robustness of WING-MTL across backbone models

The WING-MTL approach was also successfully applied and verified with other backbone-based segmentation models. The DeepLabv3 model, base model recognized as a state-of-the-art (SOTA) model on the Pascal VOC 2012 dataset. The encoder captures extensive contextual information through atrous convolutions, while the decoder enhances segmentation accuracy by refining boundary delineation [34]. The Segformer model represents a cutting-edge semantic segmentation architecture that integrates the advantages of both Transformers and CNN. This model features a hierarchical Transformer-based encoder, adept at capturing long-range dependencies and global contextual information [35,39].

Table 2 presents a comparative analysis of the Dice scores for the DeepLabv3 and Segformer models under both the STL and WING-MTL frameworks. Although overlapping confidence intervals preclude definitive statistical assertions regarding differences between STL and WING-MTL, the consistently improved Dice scores across diverse wound tissue classes and backbone architectures underscore the clinically meaningful performance enhancements achieved by WING-MTL. For example, with Segformer, WING-MTL improved Dice scores from 0.550 to 0.569 in Slough and from 0.244 to 0.347 in Epithelium segmentation, with similar gains seen in Deeplabv3. These results demonstrate that, despite reduction in WS performance for Deeplabv3, WING-MTL yields clinically meaningful improvements in WTS.

**Table 2:** Quantitative comparison between STL and WING-MTL method applied to other segmentation deep learning models.

	Dice score	STL	WING-MTL
Deeplab v3 (Resnet back bone)	Granulation	0.654	**0.672**
		(0.628- 0.680)	(0.649-0.695)
	Slough	0.533	**0.570**
		(0.511-0.554)	(0.535-0.605)
	Epithelium	0.311	**0.338**
		(0.290-0.359)	(0.314-0.362)
	Necrosis	0.700	**0.713**
		(0.677-0.722)	(0.688- 0.739)
	Wound	**0.891**	0.840
		(0.886-0.897)	(0.832-0.848)
	Dice score	STL	WING-MTL
Segformer (Transformer back bone)	Granulation	0.653	**0.665**
		(0.628- 0.678)	(0.640-0.690)
	Slough	0.550	**0.569**
		(0.533-0.567)	(0.544-0.594)
	Epithelium	0.244	**0.347**
		(0.220-0.268)	(0.306-0.388)
	Necrosis	0.602	**0.651**
		(0.540-0.664)	(0.645- 0.657)
	Wound	0.805	**0.807**
		(0.799-0.811)	(0.800-0.815)

While WING-MTL led to improved Dice scores across most tissue types in both DeepLabv3 and SegFormer, a slight reduction in performance was observed for the wound task in the DeepLabv3 model. However, this decline is relatively minor and does not detract from the overall superiority of the WING-MTL method. Additionally, the more favorable confidence intervals in WING-MTL reinforce its robustness and reliability in handling complex tissue segmentation tasks, particularly in comparison to STL. These findings underscore the efficacy of WING-MTL in enhancing segmentation performance, especially in challenging tissue types, thereby establishing it as a superior method for wound and wound tissue segmentation tasks.

## Discussion

**Fig 7:**
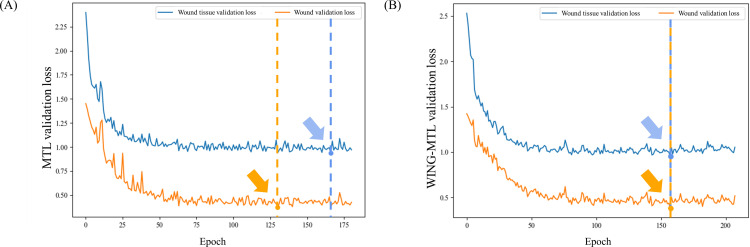
Validation loss graph of MTL and WING-MTL. (A) describes different optimization points differ for each wound and wound tissue segmentation within the MTL model, which presents a challenge for the model to achieve optimal performance for each task simultaneously. (B) shows the optimal points of each task coincide in WING-MTL. Arrows and circles indicate the convergence points of each task (orange: wound segmentation task, blue: wound tissue segmentation task).

The performance discrepancy between WS and WTS is also closely related to task imbalance, a longstanding challenge in MTL [21]. As shown in Fig 7A, analysis of the validation loss trajectories revealed that, under conventional MTL training, the WS task reached distinct convergence patterns between the two tasks. It is observed that, MTL, the segmentation of wound reached its lowest point at approximately 130 epochs, indicating optimal model performance, whereas the segmentation of wound tissue achieved its optimal point at around 170 epochs. This suggests that the wound tissue segmentation task is more complex and requires more time to learn compared to the wound segmentation task. Consequently, given that the optimization points differ for each task within the same model, it presents a challenge for the model to achieve optimal performance for each task simultaneously, and this disparity is posited as a potential cause of performance degradation. Conversely, with the proposed WING-MTL, the validation loss graph demonstrates that the optimal points for WS and WTS coincided at approximately 160 epochs (Fig 7B). This is thought to be attributed to the application of GradNorm within WING-MTL, which dynamically balances the learning rates of the tasks by adjusting the gradient norms leading to improved overall performance in the WING-MTL framework.

**Fig 8:**
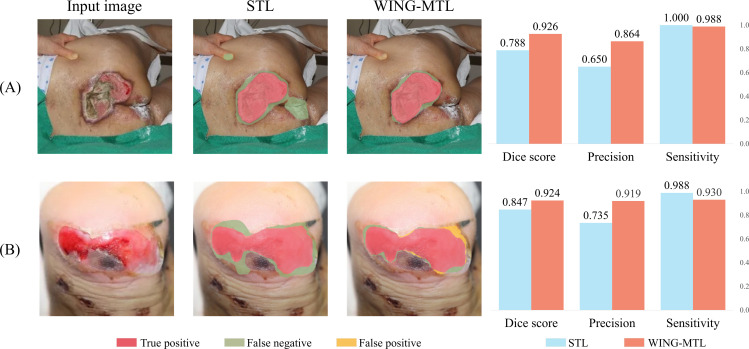
Comparison of segmentation results and quantitative metric evaluation for STL and WING-MTL. (A) Image of a pressure ulcer wound on the back of the buttocks. (B) Image of a wound on the heel.

Building on this analysis of task convergence behavior, we further examined how these differences influence quantitative performance metrics and their clinical interpretation. In this study, we examined several factors that influence the performance and reliability of wound and wound-tissue segmentation models, focusing on the differences between STL, traditional MTL, and the proposed WING-MTL framework. One notable observation concerns the interpretation of sensitivity in wound segmentation. Although STL achieved higher sensitivity than WING-MTL for the wound class in Fig 4C, closer examination of the visual results reveals that this increase is largely attributable to over-segmentation. This limitation is exemplified in Fig 8, which shows comparative segmentation results and quantitative evaluations of STL and WING-MTL models on wound images. In Fig 8A, the STL model recorded a perfect sensitivity score of 1.000, indicating detection of all positive pixels. However, visual inspection reveals that the model incorrectly classified non-wound regions such as fingernails and medication residues as wounds. Because sensitivity only accounts for false negatives, these false positives are not penalized, leading to inflated performance scores in cases of over-segmentation. However, in Fig 8B, WING-MTL shows slightly lower sensitivity but substantially higher precision and Dice scores, reflecting more balanced and clinically relevant segmentation performance. Such misclassifications and over-segmentation can result in inaccurate wound monitoring over time. Specifically, inaccurate estimation of wound size and tissue composition may introduce errors in assessing healing progress, confounding treatment response evaluation and adversely affecting clinical decision-making [18,40]. Taken together, these observations indicate that the elevated sensitivity reported for STL does not reflect true segmentation fidelity but rather the intrinsic bias of the metric toward over-segmentation. When evaluated using more clinically reliable and balanced metrics such as the Dice coefficient, WING-MTL demonstrated consistently superior performance across wound-tissue categories, with statistical analyses confirming significant improvements in all classes, as summarized in S3 Table.

Given the variability in color representation across different camera devices and acquisition conditions, we additionally conducted an experiment to evaluate whether chromatic enhancement could improve robustness in wound imaging. Color constancy and accurate color representation are fundamental challenges in medical imaging, particularly in wound analysis, where chromatic features provide essential cues for distinguishing wound-tissue types such as necrosis, slough, granulation and epithelium [19]. To address concerns, a color-temperature augmentation strategy was applied using a data-driven strategy derived from empirical histogram distribution of the training dataset, as illustrated in S3 Fig, was applied [41,42]. However, across five independent trials, this augmentation consistently reduced performance for both WS and WTS tasks, as summarized in S5 Table. Across all wound and wound-tissue categories, the integration of color temperature augmentation uniformly resulted in reduced Dice scores, demonstrating diminished algorithmic stability and compromised reliability in segmentation performance. This indicates that, despite being derived from training data statistics, the range of color perturbations introduced during enhancement may exceed the natural variability present in actual wound imaging conditions, thereby distorting diagnostically relevant color features that distinguish wound tissue types. Given that color information constitutes a critical discriminative feature for accurate WS and WTS, the application of such chromatic perturbations during training appeared to compromise ability of the model to learn stable wound tissue-specific color features. Consequently, color augmentation was intentionally excluded from the WING-MTL training pipeline to preserve optimal segmentation performance.

**Fig 9:**
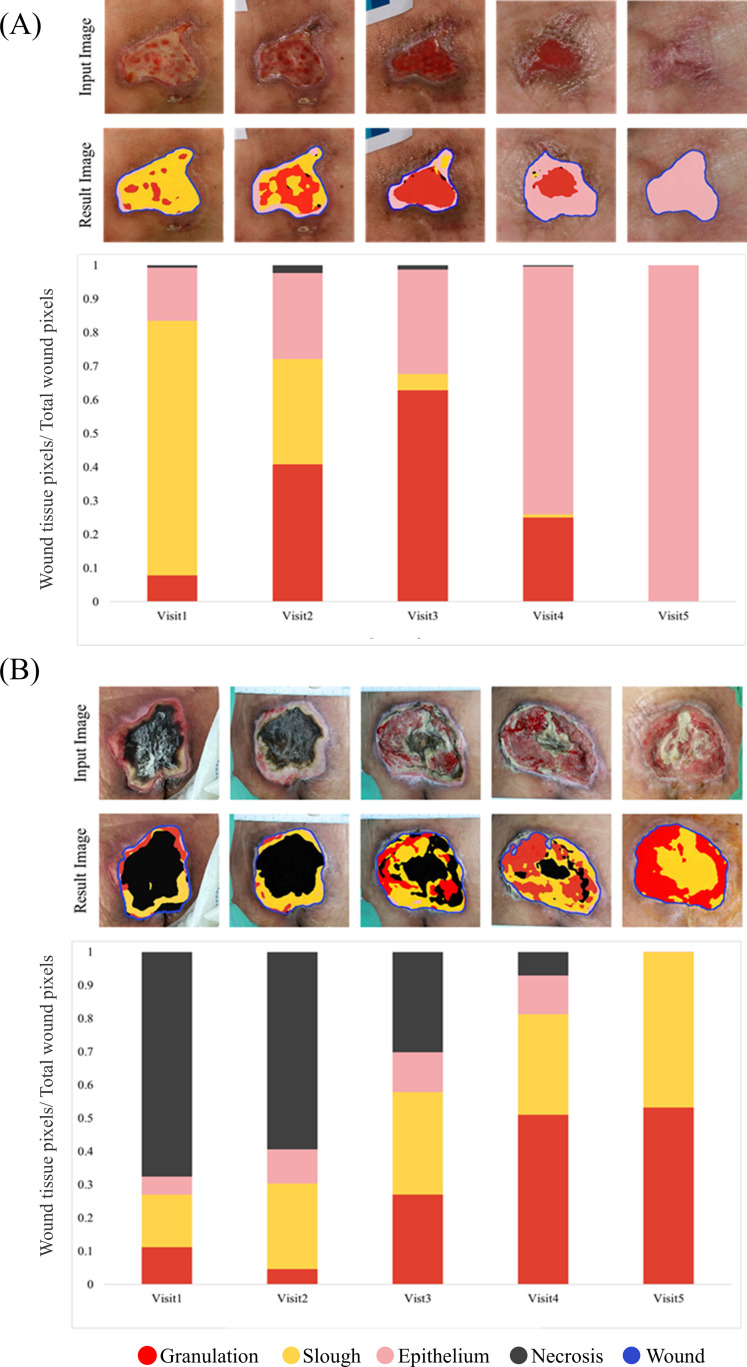
Assessment of longitudinal patients. Each wound tissue represents the proportion of wound tissue pixels relative to the total wound pixels in image taken at different visit dates. The result image displays the input image overlaid with the wound and wound-tissue segmentation outputs generated by WING-MTL.

To further examine the practical utility of WING-MTL, we evaluated its applicability for longitudinal wound monitoring. In this analysis, the proportion of wound tissue pixels relative to the total wound area was measured across different clinical visit dates, and the resulting temporal progression is shown in Fig 9. Fig 9A represents a patient who underwent five hospital visits over approximately one month. During this period, wounds and tissues were concurrently monitored using the WING-MTL framework. Initially, necrosis occupied a significant portion of the wound, but as treatment progressed, the wound tissue composition shifted towards slough and granulation, as evidenced by the graph. Fig 9B tracks the healing process of another patient over approximately two weeks. The graph shows that wound tissue initially dominated by slough transitioned significantly into granulation and epithelium, allowing for the observation of the recovery trajectory of the patient. When analyzing variations in both wound area and wound tissue composition throughout the treatment regimen, it is observed that after the initial consultation, there is often an increase in the wound size, necrotic tissue, and granulation tissue, which eventually declines following treatment. Conversely, there is an increase in granulation and epithelium tissues, indicative of wound healing progress. This simultaneous monitoring of wounds and wound tissues serves as a diagnostic aid in understanding the patient’s recovery trajectory.

## Limitations

Our study has certain limitations. First, the dataset utilized in this research was restricted to patients from Yonsei University Wonju College of Medicine, which may impact both the robustness and generalizability of the model when applied to individuals from diverse ethnic backgrounds or varied clinical environments. Therefore, the findings of this work should be interpreted as preliminary rather than conclusive evidence of clinical utility. As a critical next step, validation using larger, multi-center, and prospectively collected datasets will be necessary to establish the true robustness and applicability of the proposed framework before considering any form of clinical deployment or integration as a decision-support system. In future efforts, we plan to construct a more diverse dataset encompassing populations and settings that will enhance the model’s adaptability and reliability.

Second, the approach presented in this study employs patch-free images to develop WS and WTS algorithms, which offers significant convenience for both patients and clinicians. However, this methodology has inherent limitations in achieving precise quantification of both the wound area and its individual tissue components. In subsequent investigations, we aim to improve the accuracy of wound quantification by leveraging 3D image reconstruction techniques to transform wound images and facilitate precise volume calculations using patch-based analysis.

Third, WING-MTL demonstrated clear and consistent improvements in WTS across multiple classes and network backbones, underscoring its advantage over STL in these tasks. However, when applied to wound segmentation using the DeepLab v3 backbone, a decrease in Dice score was observed in Table 2. This suggests that while WING-MTL is highly effective for wound tissue-level segmentation, its benefits may not uniformly extend to wound-level segmentation across all architectures. We acknowledge this limitation and plan to address it in future work by investigating backbone-specific adaptations and refined task-balancing strategies to further enhance the robustness and generalizability of the method for both wound and wound tissue segmentation tasks.

## Conclusions

This study presents a comprehensive analysis of Multi-Task Learning (MTL) approaches for wound and wound tissue segmentation, emphasizing the limitations of traditional MTL frameworks, particularly regarding negative transfer issues. To overcome these challenges, we propose WING-MTL, an innovative framework that combines the parameter efficiency of MTL with improved accuracy over Separate Task Learning (STL). The WING-MTL framework effectively addresses inconsistencies observed in STL-segmented wounds and wound tissue areas, as identified through qualitative evaluations. Furthermore, we demonstrate the robustness of WING-MTL by applying it across three distinct segmentation architectures—UNet-based, ResNet-based, and Transformer-based—highlighting its adaptability and reliability. In addition, we perform a longitudinal analysis of patient data to track changes in each wound tissue type relative to wound area over the hospitalization period. This analysis reinforces the potential of WING-MTL as a valuable diagnostic tool for monitoring wound healing, providing clinicians with enhanced support for evaluating wound progression and treatment outcomes.

## Supporting information

S1 Fig The Graphic user interface-based annotation program used for wound tissue labeling. (TIF)

S2 Fig Validation loss versus parameter *α.* (TIF)

S3 Fig Data-driven color-temperature experiment. (A) Distribution of correlated color temperature in train set. (B). Representative wound images augmented using the random color augmentation.
(TIF)

S1 Table Dice score comparison of STL and MTL on wound and wound tissue segmentation tasks. (DOCX)

S2 Table Evaluation of segmentation outcomes across three Unet-based deep learning models. (DOCX)

S3 Table Statistical analysis of Dice score performance between STL and WING-MTL. Results are reported for five wound tissue classes: granulation, slough, epithelium, necrosis, and wound. Statistical significance is annotated as follows: * indicates Wilcoxon signed-rank test (p < 0.05), + indicates paired t-test (p < 0.05), and ++ indicates paired t-test (p < 0.01).
(DOCX)

S4 Table Quantitative evaluation of MTL methods based on precision, and sensitivity. (DOCX)

S5 Table Quantitative results comparing segmentation performance of WING- MTL with and without color augmentation. (DOCX)
