# Supplementary figures and images for: Development of a multi-task learning framework with gradnorm for precise wound tissue analysis
Source: PLoS One. 2026 Feb 12;21(2):e0340258. doi: 10.1371/journal.pone.0340258 (PMC12900374; doi:10.1371/journal.pone.0340258)

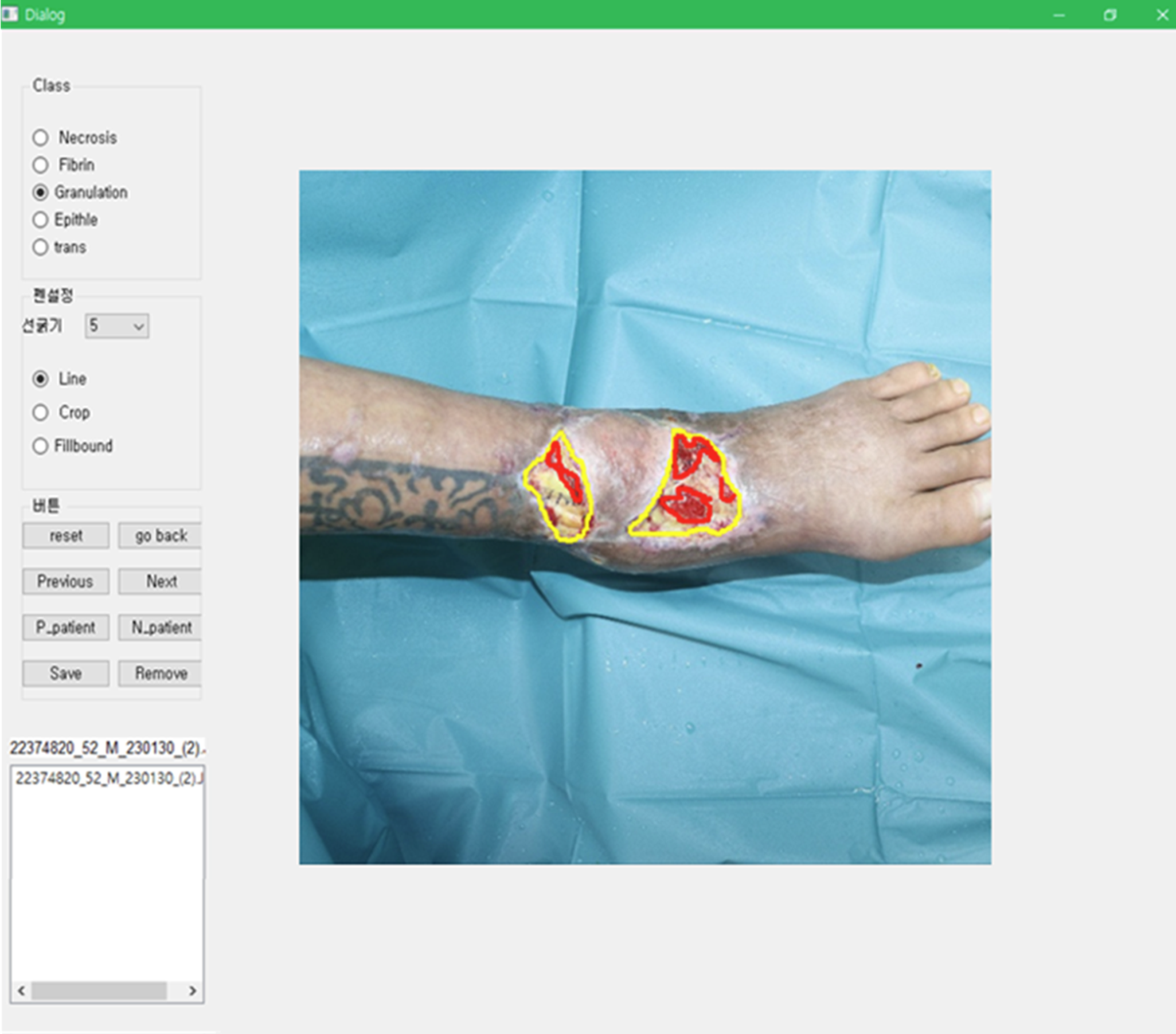

Supplement: S1 Fig — (TIF) [file pone.0340258.s001.tif]

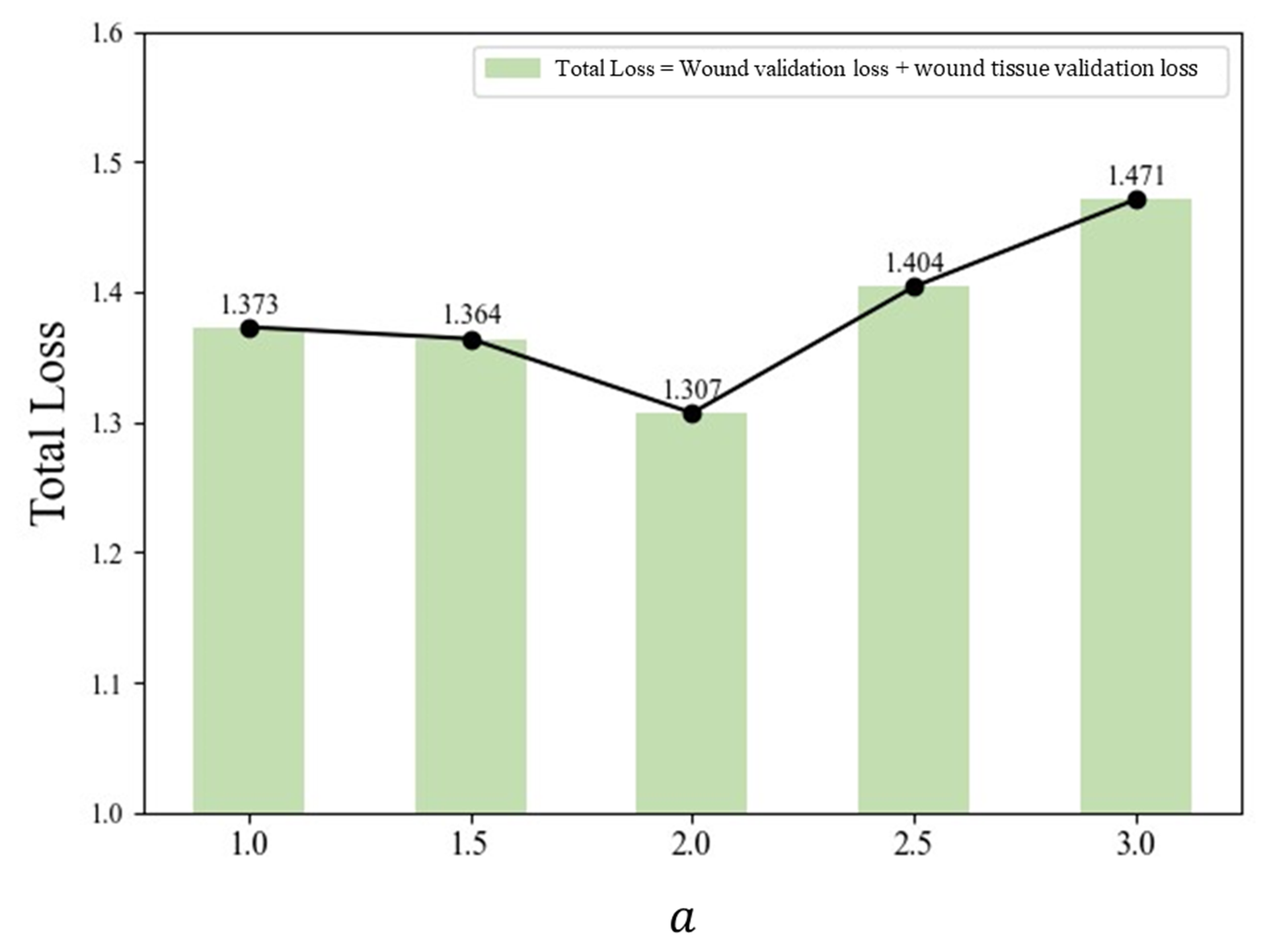

Supplement: S2 Fig — (TIF) [file pone.0340258.s002.tif]

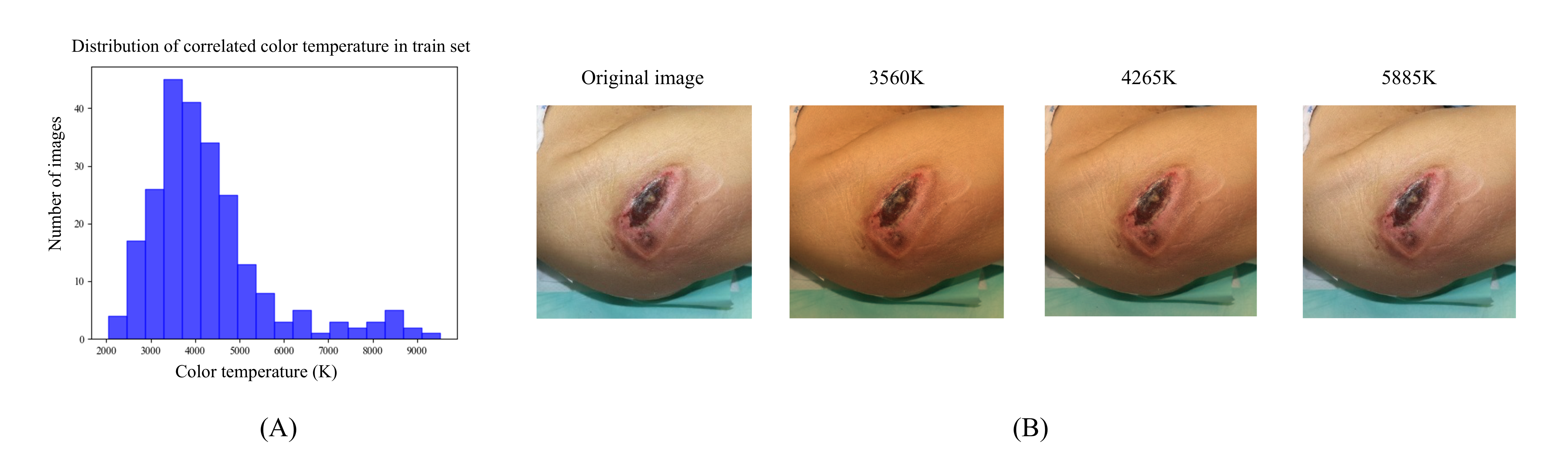

Supplement: S3 Fig — (A) Distribution of correlated color temperature in train set. (B). Representative wound images augmented using the random color augmentation. (TIF) [file pone.0340258.s003.tif]
