# Supplementary material for: Development of a multi-task learning framework with gradnorm for precise wound tissue analysis
Source: PLoS One. 2026 Feb 12;21(2):e0340258. doi: 10.1371/journal.pone.0340258 (PMC12900374; doi:10.1371/journal.pone.0340258)
Supplement: S1 Table — (DOCX) [file pone.0340258.s004.docx]

**S1 Table. Dice score comparison of STL and MTL on wound segmentation and wound tissue segmentation tasks.**

|  | Granulation | Slough | Epithelium | Necrosis | Wound |
| --- | --- | --- | --- | --- | --- |
| STL | 0.675 | **0.627** | **0.302** | **0.699** | 0.844 |
| MTL | **0.703** | 0.620 | 0.296 | 0.694 | **0.851** |

The comparison between STL and MTL using the Attention U-Net model revealed performance degradation in Slough, Epithelium, and Necrosis segmentation. This indicates that MTL may suffer from task imbalance, where prioritizing wound segmentation (WS) adversely impacts wound tissue segmentation (WTS) accuracy. These findings underscore the inherent challenge of managing task prioritization within MTL frameworks.
