# Supplementary material for: Development of a multi-task learning framework with gradnorm for precise wound tissue analysis
Source: PLoS One. 2026 Feb 12;21(2):e0340258. doi: 10.1371/journal.pone.0340258 (PMC12900374; doi:10.1371/journal.pone.0340258)
Supplement: S2 Table — (DOCX) [file pone.0340258.s005.docx]

**S2 Table. Evaluation of segmentation outcomes across three Unet-based deep learning models.**

| \|  \| Precision \| \| \| Dice Score \| \| \| \| --- \| --- \| --- \| --- \| --- \| --- \| --- \| \| Unet \| Eff-Unet \| Attention Unet \| Unet \| Eff-Unet \| Attention Unet \| \| Granulation \| 0.707 \| 0.728 \| **0.737** \| 0.673 \| 0.652 \| **0.675** \| \| Slough \| 0.563 \| 0.525 \| **0.600** \| 0.560 \| 0.472 \| **0.627** \| \| Epithelium \| **0.350** \| 0.279 \| 0.307 \| 0.295 \| 0.220 \| **0.302** \| \| Necrosis \| 0.696 \| 0.707 \| **0.716** \| 0.616 \| 0.560 \| **0.699** \| \| Wound \| 0.784 \| 0.703 \| **0.799** \| 0.836 \| 0.784 \| **0.844** \| |
| --- | --- | --- | --- | --- | --- | --- | --- | --- | --- | --- | --- | --- | --- | --- | --- | --- | --- | --- | --- | --- | --- | --- | --- | --- | --- | --- | --- | --- | --- | --- | --- | --- | --- | --- | --- | --- | --- | --- | --- | --- | --- | --- | --- | --- | --- | --- | --- | --- |
