# Supplementary material for: Development of a multi-task learning framework with gradnorm for precise wound tissue analysis
Source: PLoS One. 2026 Feb 12;21(2):e0340258. doi: 10.1371/journal.pone.0340258 (PMC12900374; doi:10.1371/journal.pone.0340258)
Supplement: S3 Table — Results are reported for five wound tissue classes: granulation, slough, epithelium, necrosis, and wound. Statistical significance is annotated as follows: * indicates Wilcoxon signed-rank test (p < 0.05), + indicates paired t-test (p < 0.05), and ++ indicates paired t-test (p < 0.01). (DOCX) [file pone.0340258.s006.docx]

**S3 Table.** Statistical analysis of Dice score performance between STL and WING-MTL across Dice score. Results are reported for five wound tissue classes: granulation, slough, epithelium, necrosis, and wound. Statistical significance is annotated as follows: * indicates Wilcoxon signed-rank test (p < 0.05), + indicates paired t-test (p < 0.05), and ++ indicates paired t-test (p < 0.01).

| \|  \| Dice score \| \| \| --- \| --- \| --- \| \| STL \| WING-MTL \| \| Granulation \| 0.675 \| **0.712** * ++ \| \| Slough \| 0.627 \| **0.655** * ++ \| \| Epithelium \| 0.302 \| **0.324** + \| \| Necrosis \| 0.699 \| **0.733** * + \| \| Wound \| 0.844 \| **0.856** * + \| |
| --- | --- | --- | --- | --- | --- | --- | --- | --- | --- | --- | --- | --- | --- | --- | --- | --- | --- | --- | --- | --- |
