# Supplementary material for: Development of a multi-task learning framework with gradnorm for precise wound tissue analysis
Source: PLoS One. 2026 Feb 12;21(2):e0340258. doi: 10.1371/journal.pone.0340258 (PMC12900374; doi:10.1371/journal.pone.0340258)
Supplement: S4 Table — (DOCX) [file pone.0340258.s007.docx]

**S4 Table. Quantitative evaluation of MTL methods based on precision, and sensitivity.**

|  | Precision | | | Sensitivity | | |
| --- | --- | --- | --- | --- | --- | --- |
|  | MTL | Nash-MTL | WING-MTL | MTL | Nash-MTL | WING-MTL |
| Granulation | 0.680 | 0.720 | **0.748** | 0.652 | 0.671 | **0.710** |
|  | (0.664-0.729) | (0.701-0.739) | (0.729-0.767) | (0.631-0.692) | (0.638-0.708) | (0.684-0.736) |
| Slough | 0.639 | 0.600 | **0.643** | 0.624 | 0.642 | **0.720** |
|  | (0.616-0.664) | (0.570-0.628) | (0.624-0.662) | (0.576-0.672) | (0.600-0.684) | (0.692-0.741) |
| Epithelium | 0.327 | 0.410 | **0.414** | 0.230 | 0.261 | **0.333** |
|  | (0.302-0.348) | (0.340-0.480) | (0.384-0.444) | (0.182-0.278) | (0.188-0.330) | (0.313-0.354) |
| Necrosis | 0.714 | 0.662 | **0.765** | 0.690 | 0.704 | **0.730** |
|  | (0.661-0.776) | (0.542-0.782) | (0.738-0.792) | (0.640-0.741) | (0.606-0.802) | (0.682-0.778) |
| Wound | 0.834 | 0.821 | **0.837** | 0.862 | 0.883 | **0.886** |
|  | (0.819-0.851) | (0.804-0.836) | (0.816-0.858) | (0.857-0.868) | (0.846-0.920) | (0.884-0.889) |
