# Supplementary material for: Development of a multi-task learning framework with gradnorm for precise wound tissue analysis
Source: PLoS One. 2026 Feb 12;21(2):e0340258. doi: 10.1371/journal.pone.0340258 (PMC12900374; doi:10.1371/journal.pone.0340258)
Supplement: S5 Table — (DOCX) [file pone.0340258.s008.docx]

**S5 Table. Quantitative results comparing segmentation performance of WING- MTL with and without color augmentation.**

|  | Granulation | Slough | Epithelium | Necrosis | Wound |
| --- | --- | --- | --- | --- | --- |
| WING-MTL  w/ coloraug | 0.641 | 0.622 | 0.318 | 0.669 | 0.848 |
| WING-MTL  w/o coloraug | **0.712** | **0.656** | **0.324** | **0.733** | **0.855** |
